# Unsupervised Screen Exposure and Poor Language Development: A Scoping Review to Assess Current Evidence and Suggest Priorities for Research

**DOI:** 10.7759/cureus.56483

**Published:** 2024-03-19

**Authors:** Georgios Korres, Melina Kourklidou, Giorgos Sideris, Despoina Bastaki, Aikaterini Demagkou, Maria Riga, Panagiotis Gogoulos, Thomas Nikolopoulos, Alexander Delides

**Affiliations:** 1 2nd Otolaryngology Department, Attikon University Hospital, National and Kapodistrian University of Athens School of Medicine, Athens, GRC; 2 First Department of Pediatrics, Unit of Developmental and Behavioral Pediatrics, Aghia Sophia Children's Hospital, Athens, GRC; 3 Department of Otorhinolaryngology-Head and Neck Surgery, Dammam Medical Complex, Dammam, SAU

**Keywords:** cognitive development, language development, language delay, screen time, screen exposure

## Abstract

Screen exposure has both negative and positive effects on the level of language skills a child acquires. The purpose of this review is to address current literature on the possible relationship between unsupervised screen exposure and language development in children and to provide recommendations to caregivers regarding screen exposure of children, taking into consideration the possible effects. A scoping review was conducted using the PubMed/MEDLINE (Medical Literature Analysis and Retrieval System Online) database. A total of 590 articles were retrieved and considered for inclusion. Twenty-one articles were finally included and reviewed with an emphasis on language, communication, and executive skills as well as cognitive development. The negative effects of screen exposure for children outweigh the positive effects. The largest number of studies demonstrate that unsupervised screen exposure may negatively impact a child's language usage and cognitive and executive skills, disrupt playtime, and affect the quality of sleep. On the other hand, supervised screen use is associated with improved language skills. More evidence is needed on unsupervised exposure in children to new types of screens. As technology could play a significant role in schools in the future, additional research is required to create educational media for schoolchildren with specific guidelines.

## Introduction and background

Attributed to the rapid development of technology over the course of the last few decades, a new phenomenon has emerged in the new era known as the digital age: screen exposure. In addition to more traditional forms of media such as television, new forms of screen technology such as tablets, mobile phones, and e-readers have emerged as crucial instruments in the everyday lives of families [1,2]. With these new user-friendly technologies, screen exposure has become more frequent, not only for adult users but also for children, lowering the age at which screen consumption begins while extending the duration of exposure [2,3]. This rise has generated discussions regarding the impact it has on the different aspects of children’s health and well-being [4].

In a cross-sectional study from Canada, 22.4% of 18-month-old children used mobile screens for a daily average of 15.7 minutes, a duration that has been increasing over the last decades [1]. In a French study, conducted in 2018, 84% of two-year-olds watched television at least once weekly, with 68% watching daily [5]. According to a nationwide research conducted in the United States in 2020, 48% of all children aged 0-8 years have their own mobile media device [6]. Mobile screen exposure has climbed over the previous decade for the same population group, from five minutes and 15 minutes daily in 2011 and 2013, respectively, up to 48 minutes of daily exposure in 2017 and 55 minutes in 2020 [6]. Most of this time is spent watching online videos and television followed by mobile gaming [6]. However, regardless of the content viewed, most studies report that television exposure is associated with worse cognitive performance [7].

It is known that language is one of the earliest and most significant indicators of a child's level of development [8]. The first three years of life are considered to be the most crucial for mental development since, during this time, a person's ability to speak and communicate with others is rapidly developed [9]. A review of recent literature on screen time and children's language development reveals contradictory findings, with both positive and negative consequences. Various factors play a key role in this association, including screen time duration, co-viewing, video characteristics, and language spoken [10,11].

The World Health Organization (WHO) suggests that children under the age of two should not be exposed to screens in any capacity and that children aged two to four years should limit their sedentary screen time to less than one hour per day [12]. According to the American Academy of Pediatrics (AAP), children younger than two years should not be exposed to any kind of screen media, with the exception of video chatting, while children aged two to five years are advised to an hour or less of high-quality viewing per day [6,9]. Guidelines for children older than six years recommend quality and quantity limitations to screen time [1,13,14].

Screen time is identified as the duration of time spent in front of any screen, either active or passive (i.e. background TV) [10]. Screen exposure may be divided into two categories: unsupervised or child-directed watching, in which the caregiver has no control over the programs watched and does not participate in the exposure, and supervised or adult-directed viewing, meaning programs specifically chosen by the caregiver for the child, along with co-viewing that allows for interactions during the exposure [10,15]. Unsupervised viewing raises more concerns as to whether it benefits or hinders a child’s language development, compared to supervised viewing [10,15].

According to current literature, screen exposure may affect different aspects of a child’s development in a variety of ways, depending on multiple factors [16]. Language, communication, executive skills, and cognitive development are some of the most commonly researched topics [1,7,10,13,14]. 

For this reason, guidelines are required in order to provide caregivers with recommendations on the amount of screen time that children should have, taking into consideration the potential effects. The purpose of this scoping review is to investigate the existing body of research on the connection between unsupervised screen time and a child's language development, as well as to identify areas of future research on this issue.

## Review

A literature research was carried out using the PubMed/MEDLINE (Medical Literature Analysis and Retrieval System Online) database to identify existing studies relevant to this topic. Four separate authors independently conducted the literature search, study selection, and data extraction for this review in May 2023. Our search was limited to articles written in English that used the key terms: ((((screen) OR (screen time)) AND (speech delay)) OR (screen time)) AND (language skills)) AND (children), (((speech delay) OR (language skills)) AND (screen time)) AND (children), (((speech)) OR (language)) AND (screen exposure) AND (children) and (((internet) OR (media)) AND (speech)) AND (children).

**Figure 1:**
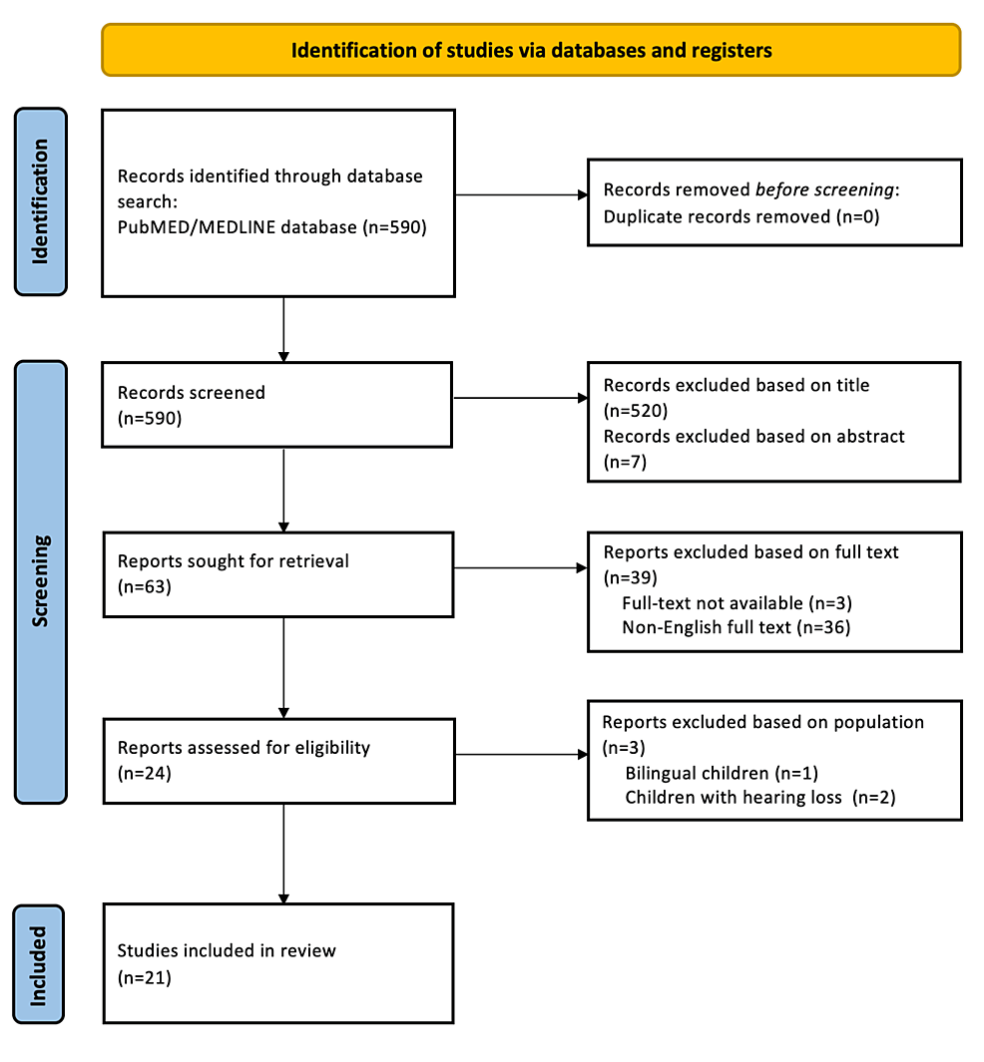
PRISMA flow chart PRISMA: Preferred Reporting Items for Systematic Reviews and Meta-Analyses

The aforementioned terms were identified in 590 articles. Articles were screened and retrieved during the search strategy using the Preferred Reporting Items for Systematic Reviews and Meta-Analyses (PRISMA) guidelines (Figure 1). The studies included in this review were mainly reviews, systematic reviews, and/or meta-analyses and original studies. Articles pertaining to bilingual children and children with hearing loss were excluded. Also excluded were articles whose full text was inaccessible or written in a language other than English.

A total of 21 articles were included in this scoping review (Table 1) [1,4,7,10,13-15,17-30]. The full-text articles were reviewed with emphasis on the possible association of unsupervised screen exposure with language skills, academic performance, autistic spectrum disorder (ASD) symptoms, cognitive development, content characteristics, school readiness, and quality of sleep.

**Table 1:** A summary of the 21 studies included in the review

Study	Study design	Outcome
Van den Heuvel et al., 2019 [1]	Cross-sectional study	Correlation identified between mobile media use and speech delay in 18-month-old children.
Arabiat et al., 2022 [4]	Systematic review	Positive association found between interactive media exposure of children and language and executive skills, in contrast with negative or neutral association to motor skills.
Eric, 2021 [7]	Review	Guidelines are needed for parents regarding screen exposure of children.
Karani et al., 2022 [10]	Scoping review	The negative effect of screen time on language development appears to be stronger than the positive effect in total. The influences are affected by the age of onset of viewing, duration of screen time, context, video characteristics, and supervision.
Madigan et al., 2020 [13]	Systematic review and meta-analysis	Recommendations are provided for parents to co-view and limit screen time for children, choosing mainly high-quality programs.
Adams et al., 2023 [14]	Scoping review	Association between screen time and cognitive delay is reported in some studies, but no specific connection has been identified.
Guellai et al., 2022 [15]	Review	Cognitive development of infants can be affected by screen use in a positive, neutral, or negative manner.
Chonchaiya et al., 2008 [17]	Case-control study	Delay in language development is associated with early onset of TV exposure and increased amount of viewing.
Richert et al., 2011 [18]	Review	A socially interactive approach appears to be useful in aiding learning from screen media for children.
Christakis, 2009 [19]	Review	No studies support the presence of advantages of TV exposure in early infancy, while the majority of evidence argues in favor of potential drawbacks.
Lawrence et al., 2021 [20]	Review	The use of mobile media devices excels over passive viewing, in terms of learning, due to the interaction required by children. On the other hand, it is correlated with language delay and poor self-regulation.
John et al., 2021 [21]	Cross-sectional study	Exposure of most preschool children to screen media exceeds the suggested limits. Variable supervision by parents is associated with potential cognitive delays.
Anderson et al., 2017 [22]	Supplement article	TV viewing in children younger than two years old is negatively correlated with executive and language skills. For preschool children this effect is controversial.
Harlé, 2019 [23]	Opinion paper	There is new evidence supporting a causative association between intensive early screen exposure and Autistic Spectrum Disorder symptoms in some children.
Madigan et al., 2019 [24]	Cohort study	Increased amounts of screen time at 24 and 36 months were significantly associated with developmental delay.
Kappos, 2007 [25]	Review	Screen media exposure has a negative impact on children’s physical and mental abilities.
Duch et al., 2013 [26]	Systematic review	Screen media use and its impact on children’s development is multifactorial, with some demographic variables more strongly linked with increased exposure and its subsequent effects.
Rithipukdee et al., 2022 [27]	Cohort study	This study concludes that a decrease in screen exposure on a daily basis will promote language development in children.
Radesky et al., 2016 [28]	Review	Exposure to appropriate TV programs and other educational media might be of benefit to preschool children, while younger children require interaction with parents during viewing. Pediatric providers play an important role in guiding caregivers in terms of screen viewing for children.
Hurwitz, 2019 [29]	Meta-analysis	Ready To Learn (RTL) initiative’s media exposure has a positive effect on children’s language skills.
Ricci et al., 2022 [30]	Systematic review	Supervision of parents and caregivers is essential during children’s exposure to the internet, as it is related to both benefits and risks.

Language skills

A systematic review and meta-analysis, including studies published over a period of 60 years, explored the associations between screen use and language skills, in terms of quantity, quality, and onset of screen use. As stated in the review, a greater amount of screen time, regardless of active or passive exposure, was associated with language delay, whereas better quality of screen use (educational programs, co-viewing) was linked with better language skills. This positive effect is also witnessed at a later age of onset of screen use [10,13]. However, young children exposed to screens do not engage in verbal dyadic interactions that have been shown to encourage communication and linguistic development [13]. A cross-sectional study that included 893 18-month-old children showed that an increase in mobile media device use of 30 minutes per day was related to a 2.3 times greater risk of expressive speech delay, as reported by parents [1]. In addition, the duration of unsupervised TV screen use in children younger than three years is also linked with difficulties in syntax at ages three and four [15].

Age of onset and academic performance

On one hand, an increased amount of viewing time at an early age has negative effects, whereas an older age of onset could be proven beneficial [13]. According to a case-control study from Thailand which included 166 children aged 15-48 months, infants who started watching TV before reaching 12 months of age and had more than two hours of daily screen exposure were approximately four times more likely to have language delay. This risk was multiplied by six when initial exposure was before the first year of life. Moreover, children with normal linguistic abilities had a statistically significant later onset of TV exposure and spent significantly less time watching TV than the case group [15,17]. The limit of this study was that in both groups, in a range of 90.9-94.6%, the content viewed was not classified as educational and it also included more male participants, in whom language delay is more common [17]. A study investigating the ability of children aged three to five years to learn new words from a 15-minute television program viewing, concluded that the experimental group performed better than the control group, with the performance of five-year-olds exceeding that of the three-year-olds [18,31]. 

Affecting a child’s language acquisition from an early age may lead to further language delay during school years and result in poorer academic achievement [19]. A longitudinal study evaluating the relationship between the early beginning of television exposure and cognitive delay in school years found that children's comprehension and intelligence measures suffered for every hour that they spent watching television [32].

Supervised and unsupervised viewing

According to the literature, co-viewing is strongly advisable in order to gain advantages from the exposure. In a case-control study by Chonchaiya and Pruksananonda, children who watched TV alone had 8.47 times more probability of having language delay, than those watching TV with their caregivers [17]. A scoping review by Karani et al. agrees that more than two hours daily of unsupervised TV watching increases the risk of low communication scores by 6.25 times versus adult-directed TV exposure [10]. The importance of co-viewing is supported by the fact that toddlers younger than two years are not mature enough developmentally to understand and make use of information transmitted from a device. For this reason, they require interaction with their caregivers to learn from a screen at that age [1,15,22]. Male participants seem to gain more benefit from co-viewing, as language delay is more common in this group [17]. Guellai et al. also support that supervised and interactive exposure to age-appropriate educational content is beneficial, whereas unsupervised viewing is associated with lower cognitive progress [15].

Background adult-directed TV, not age-appropriate for children, may affect negatively a child’s language usage and cognitive and executive skills and disrupt playtime, resulting in poorer vocabulary [15]. According to Karani et al., background TV in children younger than five years has a damaging impact on the above-mentioned and disrupts family members’ interactions [10]. Madigan et al.'s meta-analysis supports the argument that background TV is associated with decreased language skills, with no bias detected [13]. Moreover, it decreases the quantity and quality of language used by parents to communicate with toddlers, thus affecting the development of their linguistic ability [24].

An intriguing finding, concerning the environmental factors that regulate TV exposure, revealed that most of the screen time at home takes place when parents are present [15]. In non-parental care, the TV screen time is lesser when the child is in daycare (approximately 10 minutes per day) and more when the care is provided at the child's residence (approximately 1.5 hours per day). What is more, a negative association was shown with regard to the level of education of the caregiver and the screen time provided [15].

Intensive early screen exposure and ASD symptoms

Screen time and use of electronic devices, especially in the early stages, reduces communication between family members and elevates the risk of comorbidities, especially in children with neurodevelopmental issues. Intensive early screen exposure (IESE), defined as more than four hours of daily exposure, may have a causal relationship with ASD symptoms, termed “virtual autism” [23]. For example, excessive screen time in children with autism reduces their communication skills, isolates them from others in lack of quality eye contact, and may increase negative behaviors like hyperactivity, special interests, mannerisms, emotional problems, and tantrums temper. Evidence suggests improvement in such symptoms after minimizing the exposure [23]. Consequently, in order to differentiate between classical and virtual autism, a minimum of three screen-free months is suggested by the authors for overexposed children with ASD symptoms, along with interactive play [23].

In terms of emotional disorders, a Japanese study associated IESE at 18 months with attention and behavioral disorders at 30 months [33]. This study focused solely on TV exposure, while most studies discuss media screen exposure in general. Limitations to be taken into account, though, are lack of quality assessment of programs viewed, information based only on maternal reports, and that the Strengths and Difficulties Questionnaire (SDQ) that was used is intended for older children [33]. In another study, six-year-olds who were exposed to screens for more than an hour displayed more behavioral problems and concentration difficulties than non-users. Limitations of this study were the cross-sectional design, lack of information on the context of the exposure, and inclusion of individuals in a specific geographic area [34]. Children with such disorders were more frequently exposed to screens by parents to manage their tempers, thus creating a vicious circle [34]. 

In 2017, a Chinese study of 8900 children aged three to six years found behavioral characteristics similar to ASD, such as hyperactivity, peer difficulties, prosocial disorders, and temper tantrums, among children who watched more than two hours of TV each day [35]. Another study suggests that relatively healthy children, who were heavily exposed to media as infants, may also develop attentional problems by the age of seven [36].

Cognitive development

Regarding cognitive development in general, there is a causal relationship between screen time and child development [7]. In a longitudinal cohort study from Canada, including 2441 children, increased screen time in children at 24 and 36 months of age, is linked with lower performance scores assessing their developmental milestones at 36 and 60 months, respectively [24]. On the other hand, a scoping review by Adams et al. did not identify a directional association between screen exposure and infant cognitive harm [14]. According to their review, lower maternal education level and higher maternal screen time were associated with lower cognitive development scores, whereas higher education level resulted in later onset and decreased duration of screen consumption. Increased maternal screen consumption has also been linked with worse developmental test results for their children [14]. According to a study by Zimmerman and Christakis, there is a positive link between reading recognition and TV watching at three to five years of age, while other cognitive markers were affected negatively [32].

Content characteristics

Characteristics of the content and whether it is educational or not are also of significance. Although the effects of TV viewing on preschool-aged children seem to be debatable, studies show that children's cognitive development may be improved by the use of educational television [24]. Other characteristics of content, such as fast-paced videos, increased frame ratio, reduced dialogues, and an absence of encouragement to interact, seem to play a role in the development of language delay in children [24].

Non-interactive programs seem to be of bad influence on language expression while interactive programs affect the child in a positive manner by expanding their vocabulary [10]. Viewing content in a language other than the native one is also challenging, increasing the risk of language delay by 14.7 times, due to differences in syntax, grammar, and vocabulary [37]. On the other hand, it being a known enriching factor of neurodevelopment should also be taken into consideration, when assessing this risk.

New media screens

Apart from the traditional media, new media screens have become a necessity in everyday life, increasing the amount of exposure that both adults and children get. In a cross-sectional study from Canada that included 893 children with a mean age of 18.7 months, the correlation between mobile media device use and language delay was evaluated [1]. A significant association was observed; for each additional 30 minutes of screen time, there was a 2.3 times escalated risk of parent-reported speech delay. This finding, however, did not reflect on communication skills. On the contrary, moderators that did not substantially influence the results were sex, maternal education level, economic level, temperament, and participation year [1]. 

A systematic review of screen time levels in children under three years old and relative aspects identified a child’s age and minority groups (race/ethnicity) as contributing factors for high screen media use. Associations regarding maternal age, maternal education, and household socioeconomic level were unclear [26,27]. Further experimental research on mobile device use and child development association, suggests that mobile device use is more beneficial than a more passive exposure, such as TV or video watching, as it also allows children to play and interact with the screen [28]. In contrast, it is linked with language delay and poorer self-control, as demonstrated by other researchers [20]. More meticulous studies are needed to further examine these correlations.

Toddlers can learn more easily from touchscreen devices as opposed to conventional media but may have difficulty transferring this information to real-life objects and relationships, especially under the age of two [28]. Children acquire the abilities to talk, play, and communicate via their interactions with other children and members of the family. Even though mobile device screen time could be considered a learning tool for children, reduced parent-child interactions during its use might have the opposite result [1,28]. 

A newly emerged term, in line with the technological advances over the last decades, is "technoference" [7]. This term refers to the intrusion of digital technologies in human relationships at any age. A 2015 study showed that parental use of mobile phones during mealtimes resulted in poorer verbal and non-verbal social exchanges with their children. As a result, children exposed to such parental behaviors developed more negative tempers, associated with the lack of quality interactions [7]. E-books are another kind of technological advancement that has controversial effects on learning skills. On the positive side, they promote early literacy skills when compared to traditional book reading [1]. On the negative aspect, they could potentially divert children from learning and reduce the quality and quantity of contact between parents and their children [1]. Concerning recent advancements in educational software, such as new educational applications, there is not currently sufficient data to support either position [1].

School readiness

Discussion is ongoing on whether new technologies can be used effectively to prepare preschoolers. The United States Department of Education launched an initiative almost 20 years ago, named Ready To Learn (RTL) to encourage school readiness in two- to eight-year-olds, with positive outcomes [29]. A boost in children’s literacy skills, particularly in vocabulary and phonological concepts, was observed in those exposed to RTL material [29]. It is important though, to take into consideration the limitations of this study, since statistically significant outcomes were found by primarily RTL-funded researchers compared to non-RTL-funded scientists, introducing a possible bias [29]. Further research is needed to create educational media for junior students along with specific guidelines, aiming to prepare and assist them in their academic progress.

Quality of sleep

Excessive screen time affects the quality of sleep, by disrupting melatonin secretion due to blue-light emission, which may manifest with delayed onset of sleep, frequent awakenings, and nightmares, resulting in an abnormal sleep pattern [15,22,38]. A study by Lin et al. revealed a negative correlation between screen exposure and total and nighttime sleep for infants and toddlers alike [38]. Moreover, excess screen time promotes a sedentary lifestyle for children, reducing the time they spend on outdoor or other indoor physical activities, that otherwise help towards building a healthy sleep pattern [38]. 

These effects are more profound when mobile screen exposure is put under the microscope, as handheld devices such as mobile phones are easier and more readily available for use [7]. An English study, examining 715 children aged 6-36 months, discovered an important link between mobile phone daily usage and sleep pattern, reporting a decrease in sleep duration and a delayed onset of sleep [39]. Specifically, it was shown that night-time sleep was shortened by 26 minutes and nap time extended by 10 minutes, with each extra hour of mobile screen use during the day [39].

The general effects on children’s health and well-being are multifactorial and should all be taken into account before drawing any future conclusions. Practitioners should ensure that children’s screen exposure is moderate, in accordance with international guidelines [12,15,24]. In addition, healthcare professionals should collaborate with family caregivers to create a customized media plan for the family that is reflective of their needs and assists the family in striking a healthy balance between activities that take place in front of and away from the screen [24]. Further studies are needed in order to identify factors associated with increased screen exposure in young children, that can be used for future prevention and intervention policies.

Limitations of this review

The scope of this review was to address current literature on the potential relationship between unsupervised screen exposure and language development in children and to provide recommendations regarding screen exposure in children. The matching studies were identified through PubMed/MEDLINE database research. Due to the lack of studies focussing solely on unsupervised screen exposure, this review has some limitations. Overlapping factors have been detected in most papers, probably due to the multifactorial nature of linguistic and cognitive development. Also, the review includes only literature reviews (narrative, systematic with or without meta-analysis), excluding few but interesting independent studies that do not match these study designs. 

Future suggestions

The linguistic development of preschool children in connection to the amount of time spent in front of computers, monitors, and other electronic or digital media should be the subject of future scientific research [24]. Randomized controlled trials (RCTs) are regarded as the best method for establishing a causal link. On the other hand, when researching the effects of screen time on children, RCTs are not only impossible but also unethical [14]. For this reason, most of the studies rely upon parental reporting to collect data on the issue, introducing a possible bias. In children aged two to five years, the development of speech could be assessed, at perceptual and expressive levels, by using a validated developmental tool as the Preschool Language Scales- Fifth Edition (PLS-5) combined with the information on their daily habits and the time of daily exposure to screens and electronic media. Such information will be obtained via questionnaires designed for and given to caregivers or parents.

In children with speech delay, functional magnetic resonance imaging (fMRI) may play a key role in investigating the different brain activation patterns [40,41]. PLS-5 offers a comprehensive developmental language assessment with items that range from pre-verbal to emerging language to early literacy. It is a fast screening test that provides an evaluation of language disorders in infants, toddlers, and young children [42]. Respectively, the Digital Screen Exposure Questionnaire (DSEQ) is a useful tool that evaluates, among other things (sociodemographic, screen-time exposure and home media environment, media-related behaviors, level of physical activity, and parental perceptions), the screen time exposure in toddlers aged two to five years [43].

## Conclusions

Unsupervised screen exposure may negatively impact a child's language, cognitive, and executive skills. On the other hand, high-quality screen use of educational programs, along with adult supervision, especially for older children, is associated with improved language skills. However, the negative effects of screen exposure for children seem to outweigh the positive effects. Further research is needed to develop educational media for junior students along with specific guidelines, in order to assist in the social and intellectual progress of future generations.

## References

[1] van den Heuvel M, Ma J, Borkhoff CM (2019). Mobile media device use is associated with expressive language delay in 18-month-old children. J Dev Behav Pediatr.

[2] Reid Chassiakos YL, Radesky J, Christakis D, Moreno MA, Cross C (2016). Children and adolescents and digital media. Pediatrics.

[3] Durham K, Wethmar D, Brandstetter S (2021). Digital media exposure and predictors for screen time in 12-month-old children: a cross-sectional analysis of data from a German birth cohort. Front Psychiatry.

[4] Arabiat D, Al Jabery M, Robinson S, Whitehead L, Mörelius E (2023). Interactive technology use and child development: a systematic review. Child Care Health Dev.

[5] Berthomier N,  Octobre S (2019). Children and screens from 0 to 2 years old through Elfe cohort monitoring [Article in French]. Culture études.

[6] Rideout V, Robb MB (2024). The Common Sense Census: Media Use by Kids Age Zero to Eight. https://www.commonsensemedia.org/sites/default/files/research/report/2020_zero_to_eight_census_final_web.pdf.

[7] Eric O (2021). The negative effects of new screens on the cognitive functions of young children require new recommendations. Ital J Pediatr.

[8] Feldman HM (2019). How young children learn language and speech. Pediatr Rev.

[9] Linebarger DL, Vaala SE (2010). Screen media and language development in infants and toddlers: an ecological perspective. Dev Rev.

[10] Karani NF, Sher J, Mophosho M (2022). The influence of screen time on children's language development: a scoping review. S Afr J Commun Disord.

[11] (2016). Media use in school-aged children and adolescents. Pediatrics.

[12] Willumsen J, Bull F (2020). Development of WHO guidelines on physical activity, sedentary behavior, and sleep for children less than 5 years of age. J Phys Act Health.

[13] Madigan S, McArthur BA, Anhorn C, Eirich R, Christakis DA (2020). Associations between screen use and child language skills: a systematic review and meta-analysis. JAMA Pediatr.

[14] Adams C, Kubin L, Humphrey J (2023). Screen technology exposure and infant cognitive development: a scoping review. J Pediatr Nurs.

[15] Guellai B, Somogyi E, Esseily R, Chopin A (2022). Effects of screen exposure on young children's cognitive development: a review. Front Psychol.

[16] Rathnasiri A, Rathnayaka H, Yasara N, Mettananda S (2022). Electronic screen device usage and screen time among preschool-attending children in a suburban area of Sri Lanka. BMC Pediatr.

[17] Chonchaiya W, Pruksananonda C (2008). Television viewing associates with delayed language development. Acta Paediatr.

[18] Richert RA, Robb MB, Smith EI (2011). Media as social partners: the social nature of young children's learning from screen media. Child Dev.

[19] Christakis DA (2009). The effects of infant media usage: what do we know and what should we learn?. Acta Paediatr.

[20] Lawrence A, Choe DE (2021). Mobile media and young children's cognitive skills: a review. Acad Pediatr.

[21] John JJ, Joseph R, David A, Bejoy A, George KV, George L (2021). Association of screen time with parent-reported cognitive delay in preschool children of Kerala, India. BMC Pediatr.

[22] Anderson DR, Subrahmanyam K (2017). Digital screen media and cognitive development. Pediatrics.

[23] Harlé B (2019). Intensive early screen exposure as a causal factor for symptoms of autistic spectrum disorder: the case for «virtual autism». Trends Neurosci Educ.

[24] Madigan S, Browne D, Racine N, Mori C, Tough S (2019). Association between screen time and children’s performance on a developmental screening test. JAMA Pediatr.

[25] Kappos AD (2007). The impact of electronic media on mental and somatic children's health. Int J Hyg Environ Health.

[26] Duch H, Fisher EM, Ensari I, Harrington A (2013). Screen time use in children under 3 years old: a systematic review of correlates. Int J Behav Nutr Phys Act.

[27] Rithipukdee N, Kusol K (2022). Factors associated with the suspected delay in the language development of early childhood in southern Thailand. Children (Basel).

[28] Radesky JS, Christakis DA (2016). Increased screen time: implications for early childhood development and behavior. Pediatr Clin North Am.

[29] Hurwitz LB (2019). Getting a read on ready to learn media: a meta-analytic review of effects on literacy. Child Dev.

[30] Ricci RC, Paulo AS, Freitas AK (2022). Impacts of technology on children's health: a systematic review. Rev Paul Pediatr.

[31] Rice ML, Woodsmall L (1988). Lessons from television: children’s word learning when viewing. Child Dev.

[32] Zimmerman FJ, Christakis DA (2005). Children's television viewing and cognitive outcomes: a longitudinal analysis of national data. Arch Pediatr Adolesc Med.

[33] Cheng S, Maeda T, Yoichi S, Yamagata Z, Tomiwa K (2010). Early television exposure and children's behavioral and social outcomes at age 30 months. J Epidemiol.

[34] Hosokawa R, Katsura T (2018). Association between mobile technology use and child adjustment in early elementary school age. PLoS One.

[35] Wu X, Tao S, Rutayisire E, Chen Y, Huang K, Tao F (2017). The relationship between screen time, nighttime sleep duration, and behavioural problems in preschool children in China. Eur Child Adolesc Psychiatry.

[36] Christakis DA, Zimmerman FJ, DiGiuseppe DL, McCarty CA (2004). Early television exposure and subsequent attentional problems in children. Pediatrics.

[37] Perdana S, Medise B, Purwaningsih E (2017). Duration of watching TV and child language development in young children. Paediatrica Indonesiana.

[38] Lin Y, Zhang X, Huang Y (2022). Relationships between screen viewing and sleep quality for infants and toddlers in China: a cross-sectional study. Front Pediatr.

[39] Cheung CH, Bedford R, Saez De Urabain IR, Karmiloff-Smith A, Smith TJ (2017). Daily touchscreen use in infants and toddlers is associated with reduced sleep and delayed sleep onset. Sci Rep.

[40] Bernal B, Altman NR (2003). Speech delay in children: a functional MR imaging study. Radiology.

[41] Krafft H, Staudt M (2022). Clinical speech fMRI in children and adolescents : development of an optimal protocol and analysis algorithm. Clin Neuroradiol.

[42] Kwok E, Feiner H, Grauzer J, Kaat A, Roberts MY (2022). Measuring change during intervention using norm-referenced, standardized measures: a comparison of raw scores, standard scores, age equivalents, and growth scale values from the preschool language scales-fifth edition. J Speech Lang Hear Res.

[43] Kaur N, Gupta M, Kiran T, Malhi P, Grover S (2021). Development and evaluation of the digital-screen exposure questionnaire (DSEQ) for young children. PLoS One.

